# Angle-independent pH-sensitive composites with natural gyroid structure

**DOI:** 10.1038/srep42207

**Published:** 2017-02-06

**Authors:** Ruiyang Xue, Wang Zhang, Peng Sun, Imran Zada, Cuiping Guo, Qinglei Liu, Jiajun Gu, Huilan Su, Di Zhang

**Affiliations:** 1 State Key Laboratory of Metal Matrix Composites, Shanghai Jiao Tong University, 800 Dongchuan Road, Shanghai 200240, PR China

## Abstract

pH sensor is an important and practical device with a wide application in environmental protection field and biomedical industries. An efficient way to enhance the practicability of intelligent polymer composed pH sensor is to subtilize the three-dimensional microstructure of the materials, adding measurable features to visualize the output signal. In this work, *C. rubi* wing scales were combined with pH-responsive smart polymer polymethylacrylic acid (PMAA) through polymerization to achieve a colour-tunable pH sensor with nature gyroid structure. Morphology and reflection characteristics of the novel composites, named G-PMAA, are carefully investigated and compared with the original biotemplate, *C. rubi* wing scales. The most remarkable property of G-PMAA is a single-value corresponding relationship between pH value and the reflection peak wavelength (λ_max_), with a colour distinction degree of 18 nm/pH, ensuring the accuracy and authenticity of the output. The pH sensor reported here is totally reversible, which is able to show the same results after several detection circles. Besides, G-PMAA is proved to be not influenced by the detection angle, which makes it a promising pH sensor with superb sensitivity, stability, and angle-independence.

pH value is a significant parameter in applications ranging from environmental protection, industrial monitoring and biomedical treatment^1^. The development of a sensitive and accurate pH sensor which can be practically used in industry hence becomes an imperative and challenging task^2^. Stimulus-responsive intelligent polymer is a kind of functional materials that is able to rapidly change shape with respect to physical configuration or dimension under the influence of various external stimuli, including temperature^3^,^4^,^5^, magnetic field^6^,^7^, electricity^8^, light^9^, and pH value^10^,^11^,^12^,^13^,^14^. Among these responsive polymers, polymethylacrylic acid (PMAA), a cross-linked ionizable hydrophilic polymer^15^, is frequently used in pH-sensitive materials because it shows remarkable reversible swelling/collapse behavior caused by protonation/deprotonation mechanism with external pH change^3^. The carboxylic pendant groups of PMAA accept protons at low pH, while releasing them at high pH, resulting in electrostatic repulsion forces between the molecular chains and thus a volume expansion at high pH. The ability of pH sensitive hydrogels to exhibit varying level of volume swelling responding to environmental pH is highly appealing to biomedical applications of delivering bioactive molecules to the target, such as anti-cancer drug carriers to target tumors^10^,^16^.

Current chemical sensor technology has progressed to the point that smart polymers can change their configuration or dimension in environmental conditions in real-time^6^. However, the conformational change of pH-responsive intelligent hydrogels cannot be conveniently measured or directly demonstrated, laying obstacles to the expressiveness and accuracy of the output signal. Reliable measuring methods have to be created to enhance the practicability of such sensors by adding more visible and obvious features to sensitive intelligent polymers, so that their outstanding ability to response to stimuli can be conveniently demonstrated. As in most of the previous work, pH sensors is mainly in the form of polymer brush as a surface coating^17^, recently more and more scientists are in an attempt to transform the 2D-size pH-sensitive polymer membranes into subtle and complicate 3D-microstructures^2^,^18^.

For decades, the search for novel and optimal three-dimensional structures for sensors has never stopped, and a large number of sensitive photonic structures found in the nature or fabricated in laboratory have emerged^19^,^20^,^21^,^22^,^23^,^24^,^25^. After being amazed by optical structures found in the nature gemstones such as opals^19^,^20^, scientists’ attention has been caught by elaborate microstructures discovered in the nature^26^ recently. Optical microstructures in biology have made it possible to realize diverse optical effects, which are of great inspiration for bimimetic optical sensors. Bio-structures that are in research and development include biopolymeric layers with slightly different refractive indices in marine crustaceans^27^, bright colorful feathers of various colorful birds^28^,^29^, and various 3D architectures in scales of many species of beetles and butterflies^30^,^31^,^32^. Among these bio-structures, Morpho butterfly wing, which can display beautiful, natural-occurring iridescent colours that are produced by hindering specific frequencies of lights with periodic structures in scales^2^, is a good example of utilizing fine structures provided by amazing nature. Due to its characteristic photonic architecture, Morpho structures has been widely applied in various sensing fields, including thermal-infrared sensors^33^,^34^,^35^, vapor/solvent sensors^36^,^37^,^38^, chemical sensors^39^ and magnetophotonic response sensors^40^. Deng *et al*.^33^ has developed a much sensitive infrared sensor by modification of Morpho butterfly wings through physical deposition of gold (Au). In gas sensing field, Potyrailo *et al*. has done a series of work^36^,^37^ on vapor sensitivity of Morpho structured materials, and has reported a highly sensitive response to different individual vapors even in mixtures in the presence of a various moisture background recently. It is also reported that filled with PVA/chitosan (CTS), an electric field sensitive hydrogel, Morpho wing scales will exhibit an reversibly reaction to various external stimuli, including electric field, heat and air pressure^41^. As respect of pH-sensing, our previous work shows that assembling PMAA onto a Morpho butterfly wing template will equip the synthesized PMAA-Morpho materials a unique U-shaped pH response caused by the synergistic effect of chitosan and PMAA^42^. However, as a pH sensor, the shortcoming of a U-shaped response is that one outcome signal of peak wavelength will correspond to two pH values, leaving the detection results unconvincing.

On the other hand, the hierarchical structures in Morpho butterfly wing scales, though subtle and elaborate, show little symmetry characteristics, which makes it dependent on observation angle^43^, leaving a question open about the accuracy of its sensing properties. Gyroid structure (G), a bicontinuous phase found in butterfly *Callophrys rubi* with subtle microstructures and easy-controlled length scale, has characteristic inherent chiral helices and unique spherical symmetry network^44^. A mathematical description^45^ of G surface is presented in Equation (1):


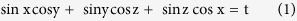


where x, y, z equals 2πX/***a***, 2πY/***a***, 2π*Z*/***a*** and (X Y Z) are the coordinates of specific points in a cubic unit cell. The values of ***a*** and ***t*** determine the size of cubic unit cell and filling volume fraction (VF) of the G networks, respectively, and VF is a determining factor of G structure’s optical properties.

Due to G structure’s unique structural characteristics, numerous G-structured functional materials have been fabricated based on gyroid bio-templates with extraordinary properties when interacting with light and matter^30^,^46^,^47^. Hsueh *et al*.^48^ has reported that metal plasmonic crystals with G structure shows ultrahigh surface plasmon resonance efficiency. What’s more, the inherent chirality of the G structure makes it possible to be employed as chiral devices^49^,^50^. Since most of the natural gyroid systems are made of organic components, research interests including combination of these G structures with functional materials to generate novel multifunctional hybrid composites^51^, and novel and convenient methods to artificially fabricate gyroid structures with an accuracy control over the size.

Herein we report a pH-sensitive hybrid material fabricated from natural gyroid structures in *Callophrys rubi* wing scales with higher sensitivity, robustness, reversibility, angle-independence and a significant single value correspondence. The hybrid composites, named G-PMAA, were achieved by deposition of PMAA (polymethylacrylic acid) onto a *C. rubi* butterfly wing. The whole materials were fabricated on the surface of glass fiber clothes, which equip the materials with high stability and strong toughness. Optical properties of G-PMAA are characteristic of its microstructure, so the external pH stimulus can be qualitatively described by its reflection features, which could be observed directly by the colour change. Further optical properties such as reflectivity and angle-dissolution contour maps are qualitatively characterized. The results show that the G-PMAA compounds have sensitive colour-tunability over pH with great uniqueness, reversibility and independence of observation angle. The presented G-PMAA compounds are superior to previous pH-sensitive composites^42^ mainly in two ways: i) stability of the test results, which is ensured by single-value correspondence between the pH stimulus and the output reflection peak wavelength; ii) independence of the measure angle due to unique spherical symmetry characteristic.

## Results and Discussion

To perform the exact angle related reflectance, it is necessary to investigate the bonding between butterfly wing and glass fiber clothes in the first place. After dried in air for 24 h, *C. rubi* wing and glass fiber clothes bind together firmly, even curving the glass fiber cloth cannot separate them. The strong bonding owes to the modification by a silane coupling agent, KH-550. KH-550 has one end of three methoxy groups (−OCH_3_), and one end of amine group (−NH_2_). After treated with ethanol, hydroxyl groups (−OH) were added on glass fiber clothes. −OCH_3_ of KH-550 hydrolyze to hydroxyl and form ether with −OH on glass fiber, while −NH_2_ will form hydrogen bonds with −NH_2_ of chitosan on alkali-treated wing scales. Thus glass fiber cloth was combined with chitosan butterfly wings successfully, and later study shows that the combination is firm enough to avoid separation during the slight volume change caused by pH variation. Combination between PMAA and the wing scales was similar to the previous research^42^. Volume fraction (VF) could be controlled by the amount adjustment of PMAA, which will thus influence reflection properties of materials.

**Figure 1:**
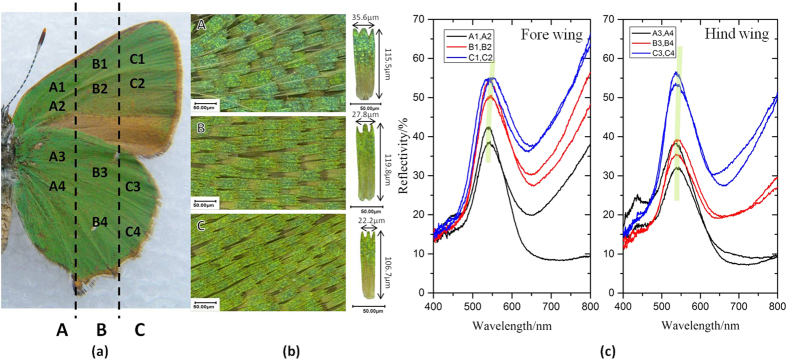
Reflection change rule of different positions in original C. rubi wing. (**a**) Photo of *Callophrys rubi* butterfly wing, divided into A, B and C zones. (**b**) 500× OM photos of each zone and an elaborate picture of the scale in the middle of the chosen area, with mean dimensions marked. (**c**) Macro reflection spectral lines of three zones in fore and hind wings.

To illustrate the colour-tunability by pH of G-PMAA, reflection curve is the most convenient and effective way due to great visualizability. Hence, reflection properties of original butterfly wing and G-PMAA after fabrication were studied respectively to investigate the reflectance change after coating. Figure 1 shows the reflection ability at different positions in original *C. rubi* wings. Both the fore wing and the hind wing are divided into three zones, named A zone, B zone, C zone, respectively, as are shown in Fig. 1(a). The optical photographs of each zone are shown in Fig. 1(b). These OM photos were taken under the magnification of 500 times, and each photo contains about 35 ± 5 wing scales. For each zone, a typical single scale was randomly picked in the middle of the zone for specified size details (800×). The measures labeled are average size of the scales in that area. It can be concluded that from A zone to C zone, wing scales become narrower and shorter, which indicates that the primary scales near the root of a wing hold larger pores than the mature ones.

In each zone, four random sites were chosen to perform the *in-situ* reflection spectra shown in Fig. 1(c). Scales near the root of the wing (A zone) are obviously greener, and even a bit bluer, than that of the scales at the end of the wings, which are far away from the root (C zone), as is shown in optical photos. This law is also confirmed by the reflectance. There is a slight red-shift of the reflection peak (539.34 nm to 541.45 nm) from A zone to C zone, illustrating that mature end of the wing reflects more long-wave light. In addition, the spectra show that fore and hind wings share the same characteristics of reflection, therefore in later study only the hind wings were chosen as representatives.

**Figure 2:**
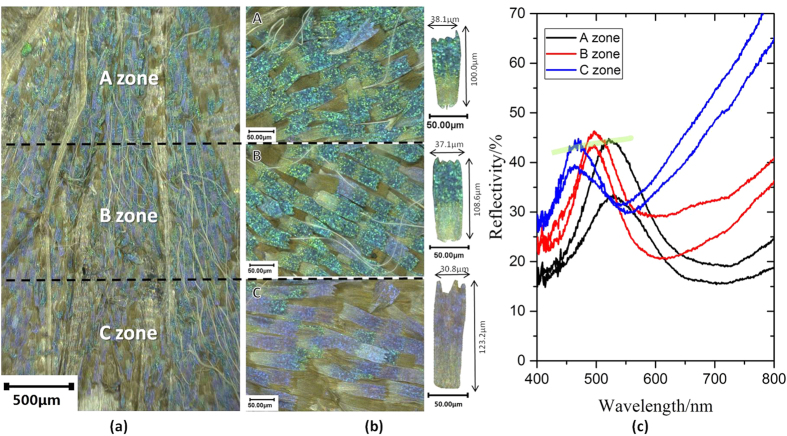
Reflection change rule of different positions in G-PMAA. (**a**) A 100× OM photo of a hind wing of G-PMAA. (**b**) 500× OM photos of A, B and C zones. (**c**) Macro reflection spectra of A, B and C zone, respectively.

The intensive optical characterization of G-PMAA is presented in Fig. 2. Similarly, each part of the wings was observed separately. It is obvious that generally gyroid structure combined with PMAA has a reflection peak at shorter wavelength compared with the original *C. rubi* wing shown in Fig. 1(c), which results in its blue-purple colour: the zone near the root of the wing shows blue in sight, but at the end of the wing it reveals obvious purple. It is not difficult to find that B zone in the middle area is a mixture of both blue scales and purple scales. Reflection spectra demonstrated in Fig. 2(c) features a distinct blue-shift of the reflection peak (523.96 nm to 463.83 nm) from A zone to C zone, totally different from original *C. rubi* wings spectra (Fig. 1a). Given that scales from A zone are larger, the pores in them are more difficult to be influenced by the filling of PMAA. On the other hand, scales from C zone are much slimmer, which enables PMAA to reconstruct the morphology by filling in the relatively small pores easier. The volume fraction (VF) changed by PMAA filling will further influence the t value in equation (1), hence changes the reflectance. Therefore, reflection curve of C zone moves the largest step towards the short-wavelength direction, resulting in an obvious blue shift from A zone to C zone.

**Figure 3:**
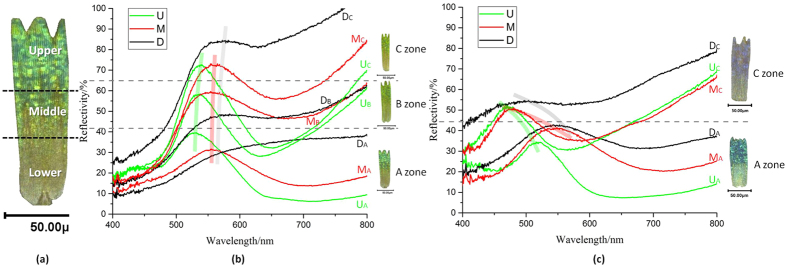
Reflection spectra of different parts in single wing scale. (**a**) illustration of reflectance positions; (**b**) reflectance in A, B and C zones of original butterfly wing; (**c**) reflectance of G-PMAA.

Due to the morphology diversity, in each scale different areas do not share consistent reflection properties either. Figure 3 further analyzes the upper, middle and lower zones in single scale of different positions of *C. rubi* wing and G-PMAA by micro-perspective spectra. Spectra of A zone and C zone in G-PMAA have more distinct reflection peaks because of the unity of scales’ colour, so only these two ends of the wing are studied. As is shown in Fig. 3(a), the tip part of a scale is defined as the upper zone (U zone), and the root part is defined as lower part (L zone), while between U and L zones is the middle zone (M zone). Table 1 lists the λ_max_ values of each spectral curve. Mostly, the tip of the scales has the highest reflectance intensity peak with the shortest wavelength value, due to the smallest pore size of gyroid structure inside. It has been convinced that larger pores will reflect longer wavelength lights with poor monochromatic^2^. In addition, gyroid structure tends to be dense and the most complete in U zone of a scale, which has strong selectivity of specific wavelength of light and thus produce the sharpest spectral curves.

Another remarkable phenomenon is that, for original rubi scales, the range of λ_max_ from upper area to lower area is approximately 35 nm. While for G-PMAA scales, the range is stenosed to about 20 nm, which means that the coating of PMAA has gathered the reflection peak of different areas in a *C. rubi* scale and made the optical features of G-PMAA more uniform and consistent. The narrowing of the peak range is beneficial for a sensing device given that it enables the consistent and accuracy of the output signal.

**Figure 4:**
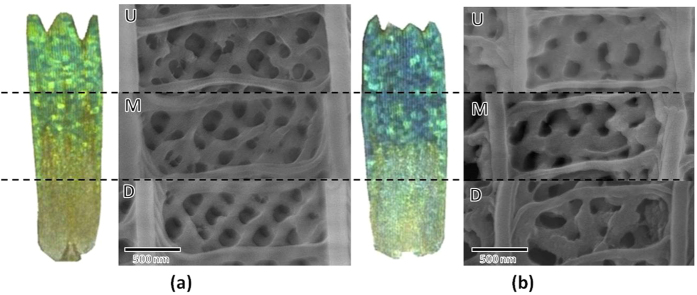
SEM pictures of upper, middle and lower parts in a scale of (**a**) original butterfly wing and (**b**) G-PMAA in A zone of the wing.

The mechanism of the narrowing phenomenon could be illustrated by the morphology characterization of the wing scales from A zone to C zone shown in Fig. 4. The mean radius of the pores in different parts of a scale has been measured from Fig. 4(a) and (b). The upper part of a scale has the smallest porous structure (143 ± 34 nm) than lower area (162 ± 37 nm) in original butterfly wing, thus it is much easier to be covered by PMAA polymer, which results in much smaller pores in G-PMAA (112 ± 17 nm). The lower part, on the other hand, whose hole size is large initially, has little change in the gyroid structure dimensions during polymerization (after coating the pore size of L zone is 142 ± 31 nm) since it is more difficult to be filled up with. This filling law is similar to that applied in the blue shift found in different positions in G-PMAA wing. To sum up, the smaller the original pore size is, the easier is the volume fraction (VF) influenced by the filling, and thus the huger change of the reflection properties caused by the t value in equation (1).

**Figure 5:**
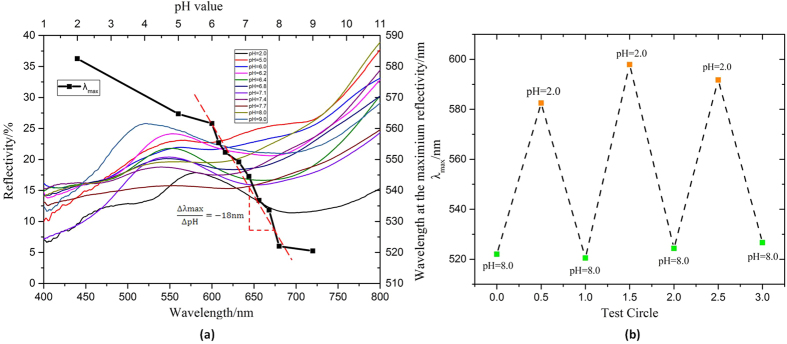
(**a**) The change of λ_max_ with different pH value. Both the reflection spectral lines and λ_max_-pH curve are included. The slope of the fitting line where pH varies from 6.0 to 8.0 is also marked to illustrate the sensibility of the pH sensor. (**b**) Evaluating the reversibility of G-PMAA pH sensor at several cyclings of the pH value.

pH-sensitivity performance of G-PMAA is measured by immersing the sample into buffer solutions with various pH values with an ionic strength deviation in a considerably small range, then testing the reflection spectra. Here, the upper part of the scales in A zone of the wing sample is chosen to be the representative because of its high reflection intensity and distinct light selectivity. The change of reflection lights with the change of pH value, as well as the spectral lines under each pH is illustrated in Fig. 5. It is easy to find that with the increase of pH value of PMAA, λ_max_ monotonously decreases. As a result, the colour of G-PMAA changes from yellow-green to green-blue with pH value changing from 2.0 to 9.0. Another significant conclusion provided by the pH-λ_max_ plot in Fig. 5 is that λ_max_ changes most rapidly with the pH value in neural condition (where pH varies from 6.0 to 8.0). This phenomenon matches well with the chemical property of PMAA, because it is proved that PMAA exhibits tremendous volume change within the pH range of 6 to 7^52^. To demonstrate the excellent accuracy and sensitivity of G-PMAA pH sensor, the distinction degree of colour is illustrated by the slope of the linear fitting line shown in Fig. 5. As a pH sensor, the reflection peak of G-PMAA changes about 18 nm with every unit change of the external pH value, which is enough to show obvious colour differential.

The remarkable pH-sensitive characteristic of G-PMAA can be explained by the swelling/de-swelling mechanism of PMAA. PMAA exhibits significant swelling or de-swelling behavior corresponding to the deprotonation of carboxyl groups inside PMAA at high pH and protonation of carboxyl groups at low pH^15^. The PMAA volume change caused by pH will determine the dimensions of gyroid structures, and hence influence the optical properties of G-PMAA. Therefore, G-PMAA has an ability to reflect the pH value of its circumstance by the wavelength of its reflection peak, which is directly observed by the colour change. The most significant advantage of G-PMAA is that its pH-sensitivity is a single value correspondence. The pH values and reflection peak wavelength are of one-to-one corresponding relationship, which will ensure the accuracy and authenticity of the test.

We also evaluated the reversibility of the pH dependency of G-PMAA, as is illustrated in Fig. 5(b). Two extreme pH values, 2 and 8 were chosen to test the stability of the reflection spectra of G-PMAA in three circles. It is shown that G-PMAA exhibits a considerably good reversibility and stability during the pH variation. Its responses to acid solution and alkalescent solution can be clearly distinguished even after several test circles. Signal drifts are illustrated by standard deviation; for pH = 2.0, the signal drift is 7.7 nm; while for pH = 8.0, the signal drift is only 2.7 nm. It could be further understand that, the optical detection of the pH sensor G-PMAA is non-destructive detection because light will do no harm to the sensor. As long as the responsive polymer PMAA is not damaged and still coated on gyroid structure, the pH sensor will keep its sensibility.

**Figure 6:**
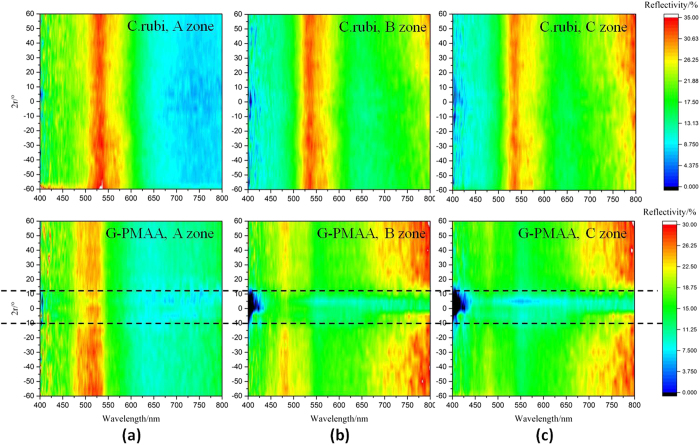
Angle dissolution reflection spectra contour map of both original *C. rubi* wing and G-PMAA. (**a**–**c**) are A, B and C zones, respectively.

As is mentioned, gyroid structure is superior to Morpho structure for its unique 3D spherical symmetry, which is insensitive to observation angle. To investigate the robustness and sensitivity to detection angle, we also tested the angle dissolution reflection of G-PMAA, as is shown in Fig. 6. The values of λ_max_ of each position matches well with the results mentioned earlier in this article. The original *C. rubi* scale shows a completely angle-independent reflection property since the reflectivity barely changes with the reflection angle of incident light in A, B and C zone. The reflectivity of G-PMAA keeps the insensitivity to observation angle since the position of λ_max_ remains constant during the change of testing direction. However, the reflection intensity is especially low when the incidence is vertical, as is marked by the dash line in Fig. 6. Such phenomenon is not conspicuous at A zone, but greatly grows when incident light moves from B zone to C zone. The morphology features of G-PMAA provides a possible reason for such change. It is certain that tilted incidence will be more efficient than vertical incidence, given that tilted incidence encounters more gyroid structures inside the scale. This law is especially valid when the hole size is small, which is caused by the coating of PMAA onto *C. rubi*. For A zone of G-PMAA, pores in scales are relatively large, so it remains independent of angle. But for B zone and C zone, the porous structures are almost filled up with PMAA, resulting in an especially low response to vertical incidence.

In spite of a relative small reflectivity with vertical lights, G-PMAA still shows characteristic robustness, given that the value of λ_max_ doesn’t changes with the testing angle. As a pH sensor, G-PMAA is competent to avoid the influence of detection angle with stable output results due to its complete gyroid structure after combination with smart polymer PMAA.

## Conclusion

To sum up, we present a promising pH sensor G-PMAA with great sensitivity, accuracy, robustness, reversibility and angle-independence, which is fabricated from natural gyroid structure. The supporting of glass fiber clothes has equipped G-PMAA with guaranteed toughness and strength. Reflection spectra of G-PMAA in both macro-perspective and micro-perspective are elaborately studied and compared with the biotemplate, *C. rubi* wing scales. It is noticed that macro-reflectivity of G-PMAA shows an obvious blue shift from A zone to C zone, in contrast to the biotemplate; and the coating of PMAA has made the reflection peak of different areas in a single wing scale more uniform and constant, which is beneficial for a pH sensor to achieve consistent output signals. G-PMAA exhibits valuable single value corresponding relationship between its reflection peak λ_max_ and pH values, which ensures the accuracy and authenticity of the sensor output. Also, the reflection peak value λ_max_ changes 18 nm with every unit deviation of pH value, showing the strong colour deferential and excellent sensitivity of the presented pH sensor. G-PMAA also exhibits good reversibility, which is able to give stable and distinguished output signals after several detection circles due to the reverse swelling mechanism of PMAA. What’s more, G-PMAA is not influenced by detection angle due to the complete gyroid structures remaining during the polymerization. There is no doubt that G-PMAA will set a good example for promising intelligent sensors fabricated from bio-structures with superb sensitivity, stability and accuracy.

Although the obtained angle-independent pH sensor fabricated from natural gyroid structures exhibits characteristic optical properties, there still leaves several limitations of this technique. First of all, making use of natural templates results in big difficulties to mass production. So far, it is still a challenge to produce well-fined G-structured materials using natural templates in a large scale. Second, the G-structured pH sensor based on organic template is not mechanically robust enough to tolerate possible stringent work conditions. Although in this article, glass fiber clothes are applied to support the composites, more improvements on the strengthening of the gyriod architecture itself should be further studied.

Based on concerning limitations of the G-structured materials fabrication technique, one of the further research directions is utilizing other fabrication methods, such as self-assembly bottom-up templates, block polymer systems (BCPs)^48^,^53^, to reduce production cost and realize industrial production. These bottom-up templates have paved a way for potential mass production of optical G-structured materials. Also top-down methods such as Direct Laser Writing (DLW)^49^,^54^ are in development for industrial production in large scale. Other development directions include fabricating G-templates with higher strength, combining other stimuli-responsive polymers with gyroid structures to make multifunctional sensors, refining the scale of the sensor particle to gain application in drug deliver field, etc. Besides, recent years the investigation into the underlying mechanisms of gyroid structure using various characterization methods has made dramatic progress, and computational-based modeling and visualizations of the G structure is rising up. It is no doubt that there is a bright future for G-structured materials lying ahead.

## Methods

### Chemicals and materials

*Callophrys rubi (C. rubi)* was purchased from the Lepidoptera Breeders Association. Glass fiber cloth was supplied by China Jushi Ltd. Ethanol, acetic acid, hydrogen chloride (HCl), sodium hydroxide (NaOH), phosphoric acid (H_3_PO_4_), sodium dihydrogen phosphate (NaH_2_PO_4_), disodium hydrogen phosphate (Na_2_HPO_4_) and methylacrylic acid (MAA) were bought from Sinopharm Chemical Reagent Co. Ltd. KH-550 cross-linking agent, ethylene glycol dimethylacrylate (EGDMA) and 2,2′-Azobis (isobutyronitrile) (AlBN) were obtained from Aladdin Chemistry Co. Ltd. MAA and EGDMA were distilled before use to remove polymerization inhibitor. Distilled water was used throughout the whole process.

### Pretreatment

First of all, a whole wing of *C. rubi* was pulled off and cleaned with water. Then it was immerged into a 6 wt% hydrogen chloride solution at 30 °C for 4 h. After that, the wing was carefully cleaned and put into a 10 wt% sodium hydroxide solution at 60 °C for 20 min, during which chitin on the surface of the original butterfly wings will be transformed to chitosan^42^. Then the wing was washed up and spread out onto a piece of pretreated glass fiber clothes. The whole sample was then kept in the dryer under 60 °C for 20 min, and left in air for 24 h. Glass fiber cloth was pretreated by steps of first being sintered at 120 °C in dryer for 2 h to get rid of organics and impurities. Then it was immerged into a 15% acetic acid solution at 80 °C for 45 min to further eliminate the organics, and treated by ethanol with ultrasonic for 20 min, during which −OH will be introduced. After that, the glass fiber was surface modified by a 10% KH-550 saline coupling agent solution to enhance the combination with butterfly wings^55^.

### G-PMAA composites preparation

MAA precursor solution is prepared by mixing 10 mL methacrylic acid monomer, 5 mL cross-linking agent EGDMA, 0.025 g AlBN as initiator and 10 mL ethanol^42^. The wing that has already been fixed on glass fiber cloth was immersed into the precursor for 24 h at room temperature, and then brought out to remove excess precursor solution with filter paper. Then the butterfly wing coated with MAA precursor was dried at 65 °C for another 24 h, resulting in polymerization on scales and finally the pH sensor G-PMAA.

### Characterization techniques

Micron-scale optical photographs of the original butterfly wing as well as G-PMAA were taken by VHX with halogen lamp (12 V 100 W) as light source. Different areas of the butterfly wing, A zone, B zone and C zone, were all observed for comparison, as is shown in Fig. 1. Nano-scale morphology of the gyroid structures of the original butterfly wing and G-PMAA were obtained by Scanning Electron Microscope (SEM, JEOL JSM-7600F), including different areas of each scale, the upper (U) zone, middle (M) zone and lower (L) zone.

Reflection properties of the original *C. rubi* wing scales and G-PMAA was tested by a Microscope Systems (ARM61) manufactured by Shanghai Ideaoptics Corporation. For macro spectra of different positions of the wings the spot size is about 150 mm. Angle resolution spectra were also performed under macro-scale, with a detection range from −60 degrees to +60 degrees. Micro spectra of different position on each scale were taken in micro-perspective with a spot diameter of 30 mm.

G-PMAA was immersed into buffer solution for 5 min to reach equilibrium, and then dried with a drier to remove excess buffer solution. Buffer solution was actually mixture of a 0.3 M H_3_PO_4_ solution, a 0.3 M NaH_2_PO_4_ solution and a 0.2 M Na_2_HPO_4_ solution according to various specific mole ratios to achieve different pH values. To eliminate the effect of ionic strength to the swelling ratio of PMAA, all of the buffer solutions are controlled in a small range of ionic strength from 0.2 to 0.6, which has little influence to the pH dependency test according to previous work on PMAA’s swelling behavior^52^. The materials went on further optical tests to obtain reflection spectra under different pH conditions. The upper zone of scales in A zone of the wing was detected to illustrate the reflection ability changing with pH value.

## Additional Information

**How to cite this article**: Xue, R. *et al*. Angle-independent pH-sensitive composites with natural gyroid structure. *Sci. Rep.* **7**, 42207; doi: 10.1038/srep42207 (2017).

**Publisher's note:** Springer Nature remains neutral with regard to jurisdictional claims in published maps and institutional affiliations.

## Figures and Tables

**Table 1:** λ_max_ of each reflection spectral line in Fig. 3.

λ_max_/nm	Original *C. rubi* scale		G-PMAA scale	
	A zone	C zone	A zone	C zone
U	529.79	538.34	519.68	470.49
M	554.64	556.96	544.55	478.31
L	564.71	573.99	545.33	493.17
